# The Role of Galcanezumab in Migraine Prevention: Existing Data and Future Directions

**DOI:** 10.3390/ph14030245

**Published:** 2021-03-09

**Authors:** Panagiotis Gklinos, Dimos D. Mitsikostas

**Affiliations:** 1 Department of Neurology, KAT General Hospital of Attica, 14561 Athens, Greece; 2 1st Neurology Department, Aeginition Hospital, National and Kapodistrian University of Athens, 11521 Athens, Greece; dmitsikostas@med.uoa.gr

**Keywords:** galcanezumab, migraine, monoclonal antibodies, migraine prevention, review

## Abstract

Galcanezumab is a humanized monoclonal antibody blocking the calcitonin gene-related peptide (CGRP) pathway by targeting the CGRP. Data from four phase-3 randomized placebo-controlled clinical trials showed that galcanezumab is superior to placebo in reducing migraine headaches, migraine-specific quality of life, and headache-related disability. Most of the adverse events (AEs) were mild to moderate and did not affect trial completion rates significantly. Along with erenumab, fremanezumab, and eptinezumab, galcanezumab forms a novel class of anti-migraine preventative treatments that is disease-specific and mechanism-based, unlike the standard ones. In addition, galcanezumab has also been shown to be effective in cluster headache, though more clinical trials are required. Overall, galcanezumab is a promising emerging treatment in migraine prophylaxis. However, it needs to be tested in larger clinical trials focused on treatment-resistant migraine. Furthermore, its safety profile, especially its potential association with an increased cardiovascular risk, needs to be established through long-term, real-world data. This review aims to give an overview of its pharmacological properties as well as to report and discuss data from clinical trials and its potential place in headache therapeutics.

## 1. Introduction

Migraine is a global disabling neurological disorder that manifests itself with recurrent episodes of headache, associated with symptoms such as nausea, photophobia, and phonophobia. Although its worldwide prevalence and socio-economic cost are well-recognized [1,2], it is still inadequately treated [3,4]. According to the American Migraine Prevalence and Prevention study (AMPP), 38% of patients with migraine should be offered preventive therapy. However, only 3–13% receive it [5,6]. Among patients who receive preventive treatment, discontinuation rates are up to 68%, mostly because of tolerability issues or lack of efficacy [7,8,9]. This is largely since until recently, preventive treatment was neither disease-specific nor mechanism-based but rather repurposed antihypertensive, antiepileptic, and antidepressant agents, therefore, causing a great number of adverse events. A recent Greek study [10] reported that 83.8% of headache participants had never taken pharmacological prophylaxis, and only 5.5% were under preventive treatment. Interestingly, a total of 61.2% of headache participants prioritized safety over effectiveness regarding prophylactic treatment.

Although migraine’s pathophysiology is not yet clear, numerous recent data indicate the crucial role of calcitonin gene-related peptide (CGRP) [11]. CGRP is 37 amino acid neuropeptide, located both in the central and the peripheral nervous system, especially in the dorsal root and trigeminal ganglions [12,13,14]. It acts as a sensory neurotransmitter, vasodilator, and mediator of neurogenic inflammation [15]. Its role in migraine pathophysiology has been implied due to studies of people with migraine, in which CGRP was found to be significantly elevated during migraine attacks, while intravenous infusion of CGRP to individuals with a history of migraine could trigger migraine attacks [12,16,17,18,19]. Besides, triptans, 5-HT1B/D receptor agonists, and migraine-specific treatments have been shown to reduce CGRP plasma levels in migraine patients [16].

Galcanezumab is a humanized monoclonal antibody (mAb) that binds CGRP and prevents its biological activity without blocking the CGRP receptor. It is administered once a month via a prefilled syringe or an autoinjector dosed at 120 mg (loading dose 240 mg). Subcutaneous galcanezumab is approved in many countries, including the USA [20] and EU countries [21], for the prevention of episodic and chronic migraine. This review aims to present a comprehensive overview of the pharmacological properties of galcanezumab as well as short- and long-term efficacy and tolerability data regarding its use.

## 2. Pharmacological Properties

### 2.1. Pharmacodynamics

Galcanezumab is a highly specific and potent humanized antibody to CGRP [22]. It binds with high affinity (K_D_ 31 pmol/L) and specificity (>10,000-fold vs. related peptides adrenomedullin, amylin, calcitonin, and intermedin) to the CGRP ligand and thereby, inhibits its binding to the receptor. It neutralizes CGRP-mediated CAMP production both in human and in rat models in a dose-dependent way. However, CAMP inhibition is not associated with prostaglandins.

Moreover, subcutaneous galcanezumab has been shown to inhibit capsaicin-induced vasodilation in vivo [23]. More specifically, in a double-blind study in healthy adults, single doses of subcutaneous galcanezumab (1–600 mg) resulted in dose-dependent inhibition of capsaicin-induced dermal blood flow, with significantly (*p* < 0.05) greater reductions with galcanezumab 75, 200, and 600 mg doses than with placebo at all post-dose timepoints (i.e., days 3, 14, 28, and 42) [23]. In a multi-dose cohort of healthy volunteers, four repeated doses of 150 mg every two weeks resulted in rapid and sustained inhibition of capsaicin-induced dermal blood flow (>175 days post-dose) [23,24].

### 2.2. Pharmacokinetics

When administered in a single subcutaneous dose, galcanezumab has exhibited dose-proportional linear pharmacokinetics across the dose range of 1–600 mg [20,24]. Pharmacokinetics of galcanezumab did not differ among healthy individuals and patients with migraine, while the site of injection did not have an impact on the absorption of the drug [21,22]. The maximum serum concentration (Cmax) of galcanezumab was approximately 30 μg/mL and the time to maximum concentration was ≈5 days. Monthly doses of 120 or 240 mg achieved a steady-state Cmax (Cmax, ss) of approximately 28 or 54 μg/mL, respectively. The galcanezumab Cmax, ss at monthly doses of 120 mg is achieved after the 240 mg loading dose. The apparent volume of distribution was 7.3 L. As a humanized IgG4 monoclonal antibody, galcanezumab is degraded into small peptides and amino acids via catabolic pathways in the same way as endogenous IgG. The apparent clearance of galcanezumab was 0.008 L/h and the elimination half-life was ≈27 days. In a population pharmacokinetics (PPK) analysis, galcanezumab pharmacokinetics were not affected by age, sex, race, migraine subtype (episodic or chronic), or bodyweight [20,21]. Additionally, it seems that renal or hepatic impairment does not affect its pharmacokinetics despite the fact that it is not tested in such patients alone. Interactions with medications that are substrates, inducers, or inhibitors of CYP450 enzymes are not likely since galcanezumab is not metabolized by these enzymes.

## 3. Efficacy

Galcanezumab has been evaluated in four randomized, double-blind, placebo-controlled, phase III, multicenter, clinical trials so far. Pivotal trials included EVOLVE-1 [25] and EVOLVE-2 [26] for the prevention of episodic migraine (4–14 migraine headache days (MHDs) per month), REGAIN [27] for the prevention of chronic migraine (≥15 headache days per month of which ≥8 were MHDs), and the most recent CONQUER [28] for the prevention of treatment-resistant episodic or chronic migraine. In EVOLVE-1, EVOLVE-2, and REGAIN trials, patients were randomly assigned to once-monthly galcanezumab 120 mg, galcanezumab 240 mg, or placebo, whilst CONQUER trial patients were assigned to once-monthly galcanezumab 120 mg or placebo. Patients who were assigned to the galcanezumab 120 mg group received a loading dose of 240 mg (two doses of 120 mg). The longer-term efficacy of galcanezumab was tested in the open-label extension arms of REGAIN [29] and CONQUER [30] studies as well as another long-term, open-label study which investigated safety, tolerability, and efficacy of galcanezumab using the same dose regimens as the EVOLVE and REGAIN studies for up to 12 months in patients with episodic or chronic migraine [31]. Efficacy data from the four pivotal double-blind, placebo-controlled clinical trials of galcanezumab are summarized in Table 1.

### 3.1. Episodic Migraine

In EVOLVE-1 and EVOLVE-2 trials, patients were included if they were aged 18–65 years with a history of episodic migraine for ≥1 year prior to enrolment, had migraine onset before the age of 50, were experiencing 4–14 MHDs per month, and had ≥2 migraine attacks per month during the baseline period. Patients were excluded if they had failed to respond to at least three classes of migraine preventive drugs, had a history of any medical or psychiatric illness that could block their participation in the study, and had prior administration of galcanezumab or any other anti- CGRP treatment. EVOLVE-1 included 858 subjects across 90 sites in North America and EVOLVE-2 studied 915 subjects at 109 study sites around the world, including sites in North America, Europe, the Middle East, and Asia. In EVOLVE-1 trial, a large proportion (60.0% of patients) had received prior preventive treatment, 18.5% had failed at least one prior treatment in the previous five years, and 4.9% had failed at least two prior preventive treatments due to lack of efficacy or poor adherence. In EVOLVE-2, 65.5% of patients had received prior preventive treatment, and 14.3% had failed at least two prior preventive treatments.

In EVOLVE-1 and EVOLVE-2, both the primary and key secondary outcomes were met [25,26]. Both galcanezumab dosages were superior in reducing the mean number of monthly MHDs compared to placebo in the six-month treatment period (primary endpoint) (Table 1). More specifically, in EVOLVE-1, the least-squares mean (LSM) reduction from baseline in monthly MHDs over the six-month treatment period was −4.7 in the 120 mg group and −4.6 in the 240 mg group, compared to −2.8 in the placebo group. Galcanezumab dosage (120 or 240 mg) did not seem to have a clinical or statistically meaningful impact. In EVOLVE-2 trial, the LSM reduction from baseline in monthly MHDs over the six-month treatment period was −4.3 in the 120 mg group and −4.2 in the 240 mg group, compared to −2.3 in the placebo group. Again, the dose itself did not affect the clinical outcome significantly. In both trials, galcanezumb administration seemed to have a rapid onset of action as patients reported a reduction in MHDs at week 1, according to a post hoc analysis [32]. The mean numbers of MHDs with acute medication use were also significantly reduced with galcanezumab 120 and 240 mg versus placebo (*p* < 0.001). Regarding other key secondary outcomes, significantly greater proportions of patients receiving galcanezumab versus placebo over the six-month treatment period demonstrated a ≥50%, ≥75%, and 100% reduction from baseline in monthly MHDs. According to the same post hoc analysis [32], a significantly greater number of patients reported a ≥50% reduction in MHDs at weeks 1–4. In addition, in both trials, patients who received galcanezumab reported fewer days of missed work or school, reduced productivity at work or school, missed household work, reduced productivity in household work, and missed family or social activities than placebo recipients. That was reflected in the significantly greater improvements in disability score (Migraine Disability Assessment (MIDAS) total score at month 6) and functioning scores Migraine-Specific Quality of Life Questionnaire (MSQ) total and subscale scores for role-function preventive, role-function restrictive (RFR), and emotional domain scores, and Patient Global Impression of Severity (PGI-S) scores.

### 3.2. Chronic Migraine

The efficacy of galcanezumab in chronic migraine was tested in REGAIN study [27], which enrolled 1113 patients with chronic migraine (≥15 headache days per month, of which ≥8 were MHDs), aged 18–65 years. Patients were excluded if they had reported persistent daily headache, cluster headache, head or neck trauma within the past six months, possible posttraumatic headache, primary headache other than chronic migraine, or prior administration of galcanezumab or other anti-CGRP treatment. Patients who had an unstable medical or psychiatric condition were also excluded from the study. The trial comprised a three-month double-blind period followed by a nine-month open-label extension.

Both dose regimens of galcanezumab were found to be superior to placebo in reducing monthly MHDs (primary endpoint). More specifically, galcanezumab 120 mg and galcanezumab 240 mg achieved a reduction of 4.8 and 4.6 monthly MHDs, respectively, compared to 2.7 achieved by placebo (*p* < 0.001). The proportion of patients who responded to treatment was significantly greater in galcanezumab 240 mg (50% and 75% response rates) and in galcanezumab 120 mg (50% response rates) compared to placebo. Regarding other key secondary outcomes, no statistically significant difference was found between galcanezumab 120 mg and placebo on reduction of monthly MHDs with acute medication use, MSQ-RFR, PGI-S score, and MIDAS score. On the other hand, besides 100% response to treatment and MIDAS score at three months, galcanezumab 240 mg was found superior to placebo on reduction of monthly MHDs with acute medication use, MSQ-RFR, and PGI-S score. No statistically significant differences were observed on any efficacy outcome between different dosages of galcanezumab.

### 3.3. Treatment-Resistant Migraine

CONQUER [28] was a multicenter, randomized, double-blind, placebo-controlled, phase 3b trial done at 64 sites in 12 countries that assessed the safety and efficacy of galcanezumab, in patients with treatment-resistant migraine (patients who had not benefited from two to four categories of migraine preventive medications). Eligible participants were 18–75 years of age with a diagnosis of migraine with aura or without aura, or chronic migraine defined by the International Classification of Headache Disorders–third edition (ICHD-3). Participants had to have failed to respond to two to four standard-of-care migraine preventive treatments due to poor efficacy or/and poor tolerance (safety reasons). Medication included antihypertensive, antiepileptic, and antidepressant agents such as propranolol, topiramate, and amitriptyline. The study comprised a three-month double-blind, placebo-controlled treatment period, and a three-month open-label extension.

Galcanezumab was found to be superior to placebo in reducing monthly MHDs (−4.1 vs. −1.0, *p* < 0.0001). The supremacy of galcanezumab was also shown in both the episodic and the chronic migraine subgroups. Regarding key secondary outcomes proportion of patients with at least 50%, 75%, and 100% reduction from baseline in monthly MHDs was significantly greater in the galcanezumab group compared with placebo in the total population (*p* < 0.0001). Finally, a significant improvement from baseline was observed with regards to functioning and disability in the galcanezumab-treated group. That was reflected in the MSQ-RFR domain score, MIDAS total score, number of monthly days with acute headache medication use in galcanezumab-treated patients compared with placebo across all study populations.

### 3.4. Open-Label Studies

In the nine-month open-label extension of the REGAIN study [29], galcanezumab administration was associated with a reduction of 6.5–7.3 monthly MHDs at six months and 8.0–9.0 monthly MHDs at 12 months. Moreover, a significant proportion reported a 50% response at six months (45%) and at 12 months (55%), whilst functioning scores (MSQ-RFR) were found to be improved.

In the open-label extension phase of CONQUER [30], galcanezumab was shown to achieve a significant reduction in monthly MHDs (−5.6 in the galcanezumab continuous group, −5.2 in those who switched from placebo to galcanezumab). Again, a great proportion of patients in both treatment groups reported a 50% response rate (54%) as well as a significant improvement in MSQ-RFR.

Finally, an open-label study investigated primarily the safety and tolerability of galcanezumab and secondarily its efficacy in patients with episodic or chronic migraine [31]. With regards to its efficacy, galcanezumab was shown to achieve a significant reduction in monthly MHDs in both dose regimens (5.6 in the 120 mg group and 6.5 in the 240 mg group). Furthermore, significant improvements were shown in the 50%, 75%, and 100% response rates, reduction of monthly MHDs with acute medication use and functioning and disability (MSQ-RFR and MIDAS score).

## 4. Safety and Tolerability

### 4.1. General Principles

Galcanezumab had a favorable safety profile and was well-tolerated, as most common treatment-emergent adverse events (TEAEs) were transient and of mild to moderate severity. These included injection-related AEs, such as injection site reaction, injection site pruritus, injection site pain and erythema, nasopharyngitis, and upper respiratory tract infection (URTI). The fact that these TEAEs were rarely associated with treatment discontinuation is of great importance for clinical practice. According to an integrated safety analysis [33], treatment discontinuation due to TEAEs was 4.0% and 3.9% in galcanezumab 120 and 240 mg treatment groups, respectively. Moreover, the proportion of patients who reported serious adverse events (SAEs) did not differ significantly between galcanezumab-treated patients and placebo groups. According to the same integrated safety analysis [33] SAEs were 2.8% and 3.0% in the galcanezumab 120 and 240 mg groups, respectively. Deaths did not occur in any of the treatment arms. Minimal changes in vital signs, laboratory values, and electrocardiograms (ECGs) were noticed among galcanezumab-treated patients, which, however, were considered neither clinically meaningful nor statistically significant. The most common adverse events (AEs) (≥2%), reported in at least one galcanezumab treatment arm of the trials as described in the integrated safety analysis of galcanezumab [33] are summarized in Table 2. Besides, the above-mentioned integrated safety analysis, galcanezumab’s favorable safety profile has also been confirmed by several published reviews and meta-analyses [34,35,36].

### 4.2. Cardiovascular Risk

Among the most important physiological properties of CGRP, is its involvement in the cardiovascular regulation of blood pressure, via vasodilation, mostly during hypertensive states rather than under normal circumstances [37]. CGRP is also considered to protect against ischemia by increasing cerebral blood flow [38], while it appears to be able to reduce post-stroke brain injury as well [39]. Finally, a case of a thalamic stroke following the first dose of another anti-CGRP antibody (erenumab) was described in a young adult, in which the most probable mechanism was vasoconstriction [40]. Therefore, the blockade of the CGRP pathway should be examined in detail both in the short and long-term.

Although none of the trials reported an increased risk of developing cardiovascular adverse events following galcanezumab administration, it has to be mentioned that in the EVOLVE-2 trial, two patients in the galcanezumab 240 mg group reported acute myocardial infarction and transient ischemic attack (TIA) while hypertension was observed in five patients. Thus, larger, long-term trials need to be conducted to confirm galcanezumab’s safety, especially regarding cardiovascular risk.

### 4.3. Immunogenicity and Neutralizing Antibodies

Sometimes, the formation of antidrug antibodies due to the recognition of mAbs as allogenic may result in mAb neutralization and rapid clearance, low efficacy, and adverse events, as well as allergic reactions and increased cost of treatment. According to a study that evaluated the immunogenicity of galcanezumab in phase-3 trials [40], the incidence of treatment-emergent antidrug antibodies was 2.6–12.4% in the galcanezumab group and 0.5–1.7% in the placebo group. Fortunately, the observed antidrug antibody titer did not affect galcanezumab concentrations, efficacy, or CGRP concentrations. Furthermore, no allergic reactions nor increased rate of ADA-related adverse events were noticed. However, it has to be highlighted that ADAs titer results may vary according to the selected assay methodology and should be interpreted carefully.

## 5. Galcanezumab and Other mAbs in Migraine Prophylaxis

The unmet need for highly effective, yet well-tolerated migraine preventive treatment led to the development of mAbs targeting the CGRP pathway. At the moment, besides galcanezumab, the agents that are currently marketed include erenumab, eptinezumab, and fremanezumab. All of them are large peptides, have high target specificity with minimal potential for off-target toxicity, are degraded and cleared within the reticuloendothelial system, do not undergo hepatic metabolism or renal clearance, and do not compete for binding sites. Therefore, there is a minimal likelihood of drug interactions [41]. In addition, as large molecules, they are administrated parenterally and do not cross the blood-brain barrier, thus minimizing central nervous system side effects and toxicity. Regarding their differences, galcanezumab, fremanezumab, and eptinezumab are humanized monoclonal antibodies, whilst erenumab is the only fully human. All of them are administered once a month subcutaneously, except from eptinezumab, which requires quarterly intravenous administration. Erenumab is the only drug that binds to the CGRP receptor whilst the other three act through binding to the ligand itself. Moreover, it has to be mentioned that all four mAbs have different constant regions (Fc) and are categorized into the following subclasses: Erenumab is an IgG2 antibody, eptinezumab an IgG1 antibody, fremanezumab an IgG2 antibody and galcanezumab an IgG4 antibody. IgG2 and IgG4 exhibit a lower affinity to the Fcγ receptor and are preferred for blocking antigen function. More specifically, both subclasses are preferred when aiming to neutralize the soluble antigen without inducing the host effector mechanisms [42]. Finally, special attention should be given to the fact that galcanezumab is the only one among the four currently approved mAbs, which has also been approved for the prevention of cluster headache [43]. The efficacy of galcanezumab for the treatment of adults with episodic cluster headache was evaluated in an eight-week, randomized, double-blind, multinational, phase 3 trial. Results showed that it reduced the frequency of weekly attacks (−8.7 at the active group vs. −5.2 at the placebo group attacks/week) and met its primary endpoint. Moreover, at week 3, 71% and 53% of patients in the galcanezumab and placebo groups achieved a ≥50% reduction in the weekly cluster headache attack frequency. Given the fact that cluster headache is an extremely painful headache disorder, galcanezumab’s efficacy in the prevention of attacks in the active phase is of great importance and might pave the way for further future research. However, there need to be more studies to establish its efficacy.

## 6. Discussion

Galcanezumab is a humanized monoclonal antibody specifically developed for migraine prophylaxis. It acts by blocking the CGRP pathway by targeting the ligand itself. In the pivotal studies [25,26], galcanezumab was shown to be efficacious in reducing monthly MHDs, monthly MHDs with acute medication use as well as improving response rates and functional and disability scores (MIDAS, MSQ R-FR). Long-term efficacy data (up to 12 months) were also shown in the open-label extension trials [29,30,31] and were also confirmed by a pooled analysis [44] and several systematic reviews and metanalyses [34,35,36]. Galcanezumab was also well-tolerated and safe, which is reflected in the high completion rates of the trials (>80%, similar to placebo) and the low drop-out rates due to AEs. Moreover, more than 75% of the participants completed the 12-month open-label extension studies, confirming the favorable profile of the studied drug in the mid-term. In terms of severity, most of the AEs were mild to moderate (most frequent AE was injection site pain), transient, and resolved during the monitoring stage. However, it has to be mentioned that galcanezumab’s long-term safety should be investigated carefully in the clinical setting, especially due to the unknown consequences of a long-term blockade of the CGRP pathway. As mentioned above, CGRP plays a crucial role in the physiological regulation of blood pressure, mostly through vasodilation. Therefore, long-term monitoring through real-world data is required to determine the potential risks of anti-CGRP monoclonal antibodies.

Monoclonal antibodies targeting the CGRP pathway include galcanezumab, fremanezumab, and eptinezumab, which target the ligand, and erenumab, which blocks the receptor. Galcanezumab is the only one shown to be efficacious in cluster headache as well [43] though, its efficacy needs to be established in more randomized clinical trials. Although no head to head trials with anti-CGRP mAbs and migraine standard prophylactic treatments have been conducted so far, according to a systematic review and a likelihood to help or harm (LHH) analysis [45] anti-CGRP mAbs had higher LHH values than propranolol or topiramate for episodic migraine and onabotulinumtoxinA or topiramate for chronic migraine prevention. Noticeably, galcanezumab had the highest LHH ratio in chronic migraine. However, in episodic migraine the agent that was more likely to be beneficial than harmful was fremanezumab.

Although combined treatments in refractory chronic migraine have not yet been studied thoroughly, the question of whether dual preventive therapy with mAbs and onabotulinumtoxinA could be beneficial for patients with refractory chronic migraine has been raised recently. Preclinical data showed that mAbs and onabotulinumtoxinA may have synergic action within the trigeminovascular system. Most significant data show that fremanezumab, prevents the activation of Aδ- but not C-fibers, whereas onabotulinumtoxinA prevents the activation of C- but not Aδ-fibers. Fremanezumab is likely effective in migraine patients with pain signals from meningeal Aδ-fibers. In contrast, non-responders may involve other pathways mediated by C-fibers and/or different central trigeminovascular neurons. Thus, concurrent use of medications blocking the activation of meningeal C-fibres may provide a synergistic effect on the trigeminal nociceptive pathway [46]. However, these data need to be established through rigorous trials assessing efficacy and safety in the clinical setting. The development of anti-CGRP monoclonal antibodies represents a very promising emerging option in the prevention of episodic and chronic migraine. Until recently, patients who met the criteria for migraine prophylaxis were treated with β-blockers, such as propranolol, oral antiepileptics, such as valproate, divalproex, topiramate, and antidepressants, such as amitriptyline and venlafaxine. However, these agents were not specifically developed for migraine, thus resulting in a noticeable number of TEAEs and poor treatment adherence. On the other hand, anti-CGRP monoclonal antibodies have a greater half-life time compared to conventional oral agents, which allows monthly administration instead of daily intake, making treatment more convenient for migraine patients and potentially improving adherence, thus efficacy.

Although clinical trials showed promising results, galcanezumab needs to be tested through real-world data in the long-term. Unfortunately, all four monoclonal antibodies, including galcanezumab, are costly treatments and cannot be afforded by many patients. Consequently, they are considered a second-line option in migraine prophylaxis and are mostly prescribed in chronic migraine and especially to patients who have failed at least two or three classes of migraine preventive treatments. In the future, studies with galcanezumab need to be focused more on treatment-resistant migraine rather than episodic, as most episodic migraineurs do not receive galcanezumab as first-line therapy.

Furthermore, migraine is frequently comorbid with other medical conditions, such as psychiatric conditions (most common) and sleep disorders. This is why most of the older oral conventional prophylactic migraine treatments were selected according to drug-drug interactions and the presence of such comorbidities. However, in the clinical trial settings, most comorbidities consisted of exclusion criteria. Hence, galcanezumab needs to be tested in more patients with comorbidities to determine its efficacy in that complex population.

## 7. Conclusions

Galcanezumab is a promising and effective emerging treatment for migraine prophylaxis. Its safety profile and monthly administration due to its greater half-life time resulted in low drop-out rates in the clinical trials and imply better treatment adherence, thus more efficacy. However, it remains a costly option when compared to older conventional agents, such as antidepressants, b-blockers, and antiepileptics. Moreover, its safety should be investigated in the long term with larger clinical trials focused on treatment-resistant migraine as well as real-world data to determine its potential association with an increased cardiovascular risk.

## Figures and Tables

**Table 1:** Efficacy of galcanezumab in migraine prevention in phase-3 double-blind clinical trials.

Outcome		EVOLVE-1			EVOLVE-2			REGAIN			CONQUER	
		Placebo	120 mg	240 mg	Placebo	120 mg	240 mg	Placebo	120 mg	240 mg	Placebo	120 mg
Change from baseline in the no. of MHDs/month		−2.8	−4.7 ^1^	−4.6 ^1^	−2.3	−4.3 ^1^	−4.2 ^1^	−2.7	−4.8 ^1^	−4.6 ^1^	−1.0	−4.1 ^1^
Percentage of patients with a reduction in MHDs/month (response rate)	50%	38.6	62.3 ^1^	60.9 ^1^	36.0	59.3 ^1^	56.5 ^1^	15.4	27.6 ^1^	27.5	13.3	37.7 ^1^
	75%	19.3	38.8 ^1^	38.5 ^1^	17.8	33.5 ^1^	34.3 ^1^	4.5	7.0 ^2,3^	8.8 ^1^	3.3	14.5 ^1^
	100%	6.2	15.6 ^1^	14.6 ^1^	5.7	11.5	13.8 ^1^	0.5	0.7	1.3	0	4.9 ^1^
Change from baseline in MIDAS total score		−14.9	−21.2 ^1^	−20.1 ^1^	−12.0	−21.2 ^1^	−20.2 ^1^	−11.5	−20.3	−17.0	−3.3	−21.3 ^1^
Change from baseline in MSQ-RFR		24.7	32.4 ^1^	32.1 ^1^	19.7	28.5 ^1^	27.0 ^1^	16.8	21.8 ^1^	23.1 ^1^	10.7	23.2 ^1^

MHDs: Migraine headache days, MIDAS: Migraine Disability Assessment, MSQ-RFR: Migraine-Specific Quality of Life Questionnaire. In the 120 mg group, patients received a 240 mg loading dose, then 120 mg. ^1^ *p* < 0.001; ^2^ *p* < 0.5; ^3^ not significant after adjustment for multiplicity.

**Table 2:** Safety profile of galcanezumab.

Adverse Events (AEs)	Galcanezumab 120 mg (*n* = 926) (%)	Galcanezumab 240 mg (*n* = 1350) (%)
Overview		
TEAEs in ≥1 patient	68.8	74.4
Drop-outs due to AEs	4.0	3.9
SAEs	2.8	3.0
TEAs (≥2%)		
Injections site pain	11.0	9.6
Nasopharyngitis	9.4	8.5
Upper respiratory tract infection	6.8	7.0
Injection site reaction	5.2	6.5
Dizziness	2.8	3.3
Injection site erythema	3.2	4.4
Sinusitis	4.1	3.9
Urinary tract infection	2.6	3.8
Influenza	2.3	4.1
Fatigue	2.2	2.7
Injection site pruritus	2.3	2.8
Cough	1.8	2.5
Oropharyngeal pain	1.9	2.1
Bronchitis	1.8	2.7
Rash	1.8	2.0

TEAEs: Treatment-emergent adverse events, SAEs: Serious adverse events.

## Data Availability

Data sharing not applicable.

## References

[1] Steiner T.J., Stovner L.J., Birbeck G.L. (2013). Migraine: The seventh disabler. Cephalalgia.

[2] GBD 2016 Neurological Disorders Collaborator Group (2017). Global, regional, and national incidence, prevalence, and years lived with disability for 328 diseases and injuries for 195 countries, 1990–2016: A systematic analysis for the Global Burden of Disease Study 2016. Lancet.

[3] Lipton R.B., Silberstein S.D. (2015). Episodic and chronic migraine headache: Breaking down barriers to optimal treatment and prevention. Headache.

[4] Peck K.R., Johnson Y.L., Smitherman T.A., Aminoff M.J., Boller F., Swaab D.F. (2016). Handbook of Clinical Neurology.

[5] Lipton R.B., Bigal M.E., Diamond M., Freitag F., Reed M.L., Stewart W.F. (2007). Migraine prevalence, disease burden, and the need for preventive therapy. Neurology.

[6] Blumenfeld A.M., Bloudek L.M., Becker W.J., Buse D.C., Varon S.F., Maglinte G.A., Wilcox T.K., Kawata A.K., Lipton R.B. (2013). Patterns of use and reasons for discontinuation of prophylactic medications for episodic migraine and chronic migraine: Results from the second international burden of migraine study (IBMS-II). Headache.

[7] Berger A., Bloudek L.M., Varon S.F. (2012). Adherence with migraine prophylaxis in clinical practice. Pain Pract..

[8] Diamond S., Bigal M.E., Silberstein S., Oster G. (2007). Patterns of diagnosis and acute and preventive treatment for migraine in the United States: Results from the American Migraine Prevalence and Prevention study. Headache.

[9] Loder E.W., Rizzoli P. (2011). Tolerance and loss of beneficial effect during migraine prophylaxis: Clinical considerations. Headache.

[10] Constantinidis T.S., Arvaniti C., Fakas N., Rudolf J., Kouremenos E., Giannouli E., Mitsikostas D.D. (2021). A population-based survey for disabling headaches in Greece: Prevalence, burden and treatment preferences. Cephalalgia.

[11] Tepper S.J. (2018). History and review of anti-calcitonin gene-related peptide (CGRP) therapies: From translational research to treatment. Headache.

[12] Goadsby P.J., Edvinsson L., Ekman R. (1988). Release of vasoactive peptides in the extracerebral circulation of humans and the cat during activation of the trigeminovascular system. Ann. Neurol..

[13] Goadsby P.J., Edvinsson L., Ekman R. (1990). Vasoactive peptide release in the extracerebral circulation of humans during migraine headache. Ann. Neurol..

[14] Goadsby P.J., Holland P.R., Martins-Oliveira M., Hoffmann J., Schankin C., Akerman S. (2017). Pathophysiology of migraine: A disorder of sensory processing. Physiol. Rev..

[15] Ho T.W., Edvinsson L., Goadsby P.J. (2010). CGRP and its receptors provide new insights into migraine pathophysiology. Nat. Rev. Neurol..

[16] Goadsby P.J., Edvinsson L. (1993). The trigeminovascular system and migraine: Studies characterizing cerebrovascular and neuropeptide changes seen in humans and cats. Ann. Neurol..

[17] Lassen L.H., Haderslev P.A., Jacobsen V.B., Iversen H.K., Sperling B., Olesen J. (2002). CGRP may play a causative role in migraine. Cephalalgia.

[18] Lassen L.H., Jacobsen V.B., Pedersen P.A., Sperling B., Iversen H., Olesen J. (1998). Human calcitonin gene-related peptide (hCGRP)-induced headache in migraineurs. Eur. J. Neurol..

[19] Hansen J.M., Hauge A.W., Olesen J., Ashina M. (2010). Calcitonin gene-related peptide triggers migraine- like attacks in patients with migraine with aura. Cephalalgia.

[20] Eli Lilly (2019). EMGALITY (galcanezumab-gnlm) Injection, for Subcutaneous Use: US Prescribing Information. http://www.fda.gov/.

[21] European Medicines Agency (2018). Emgality: Summary of Product Characteristics. http://www.ema.europa.eu/.

[22] Benschop R.J., Collins E.C., Darling R.J., Allan B.W., Leung D., Conner E.M., Nelson J., Gaynor B., Xu J., Wang X.F. (2014). Development of a novel antibody to calcitonin gene-related peptide for the treatment of osteoarthritis-related pain. Osteoarthr. Cartil..

[23] Vermeersch S., Benschop R.J., Van Hecken A., Monteith D., Wroblewski V.J., Grayzel D., de Hoon J., Collins E.C. (2015). Translational pharmacodynamics of calcitonin gene-related peptide monoclonal antibody LY2951742 in a capsaicin-induced dermal blood flow model. J. Pharmacol. Exp. Ther..

[24] Monteith D., Collins E.C., Vandermeulen C., Van Hecken A., Raddad E., Scherer J.C., Grayzel D., Schuetz T.J., de Hoon J. (2017). Safety, tolerability, pharmacokinetics, and pharmacodynamics of the CGRP binding monoclonal antibody LY2951742 (galcanezumab) in healthy volunteers. Front. Pharmacol..

[25] Staufer V.L., Dodick D.W., Zhang Q., Carter J.N., Ailani J., Conley R.R. (2018). Evaluation of galcanezumab for the prevention of episodic migraine: The EVOLVE-1 randomized clinical trial. JAMA Neurol..

[26] Skljarevski V., Matharu M., Millen B.A., Ossipov M.H., Kim B.K., Yang J.Y. (2018). Efficacy and safety of galcanezumab for the prevention of episodic migraine: Results of the EVOLVE-2 Phase 3 randomized controlled clinical trial. Cephalalgia.

[27] Detke H.C., Goadsby P.J., Wang S., Friedman D.I., Selzler K.J., Aurora S.K. (2018). Galcanezumab in chronic migraine: The randomized, double-blind, placebo-controlled REGAIN study. Neurology.

[28] Mulleners W.M., Kim B., Lainez M.J., Lanteri-Minet M., Pozo-Rosich P., Wang S., Tockhorn-Heidenreich A., Aurora S.K., Nichols R.M., Yunes-Medina L. (2020). A randomized, placebo-controlled study of galcanezumab in patients with treatment-resistant migraine: Double-blind results from the CONQUER Study (162). Neurology.

[29] Detke H., Pozo-Rosich P., Reuter U., Dolezil D., Li L.Q., Wang S., Aurora S.K. (2019). One-year treatment with galcanezumab in patients with chronic migraine: Results from the open-label phase of the REGAIN study [abstract no. P2.10-010]. Neurology.

[30] Detke H.C., Reuter U., Lucas C., Dolezil D., Hand A., Tockhorn-Heidenreich A., Stroud C., Aurora S.K. Galcanezumab in patients with treatment-resistant migraine: Results from the open-label phase of the CONQUER phase 3 trial [abstract plus poster no. 43625]. Proceedings of the American Academy of Neurology Annual Meeting.

[31] Camporeale A., Kudrow D., Sides R., Wang S., Van Dycke A., Selzler K.J., Stauffer V.L. (2018). A phase 3, long-term, open-label safety study of Galcanezumab in patients with migraine. BMC Neurol..

[32] Detke H.C., Millen B.A., Zhang Q., Samaan K., Ailani J., Dodick D.W., Aurora S.K. (2020). Rapid onset of effect of galcanezumab for the prevention of episodic migraine: Analysis of the EVOLVE studies. Headache.

[33] Bangs M.E., Kudrow D., Wang S., Oakes T.M., Terwindt G.M., Magis D., Yunes-Medina L., Stauffer V.L. (2020). Safety and tolerability of monthly galcanezumab injections in patients with migraine: Integrated results from migraine clinical studies. BMC Neurol..

[34] Zhao X., Xu X., Li Q. (2020). Efficacy and safety of galcanezumab for preventive treatment of migraine: A systematic review and meta-analysis. J. Neurol..

[35] Gklinos P., Mitsikostas D.D. (2020). Galcanezumab in migraine prevention: A systematic review and meta-analysis of randomized controlled trials. Ther. Adv. Neurol. Disord..

[36] Martin V., Samaan K.H., Aurora S., Pearlman E.M., Zhou C., Li X., Pallay R. (2020). Efficacy and safety of galcanezumab for the preventive treatment of migraine: A narrative review. Adv. Ther..

[37] Smillie S.J., King R., Kodji X., Pozsgai G., Fernandes E., Marshall N., de Winter P., Heads R.J., Dessapt-Baradez C., Gnudi L. (2014). An ongoing role of α-calcitonin gene-related peptide as part of a protective network against hypertension, vascular hypertrophy, and oxidative stress. Hypertension.

[38] Zhang J.Y., Yan G.T., Liao J., Deng Z.H., Xue H., Wang L.H., Zhang K. (2011). Leptin attenuates cerebral ischemia/reperfusion injury partially by CGRP expression. Eur. J. Pharmacol..

[39] Aradi S., Kaiser E., Cucchiara B. (2019). Ischemic stroke associated with calcitonin gene-related peptide inhibitor therapy for migraine: A case report. J. Stroke Cerebrovasc. Dis..

[40] Martinez J.M., Hindiyeh N., Anglin G., Kalidas K., Hodsdon M.E., Kielbasa W., Moser B.A., Pearlman E.M., Garces S. (2020). Assessment of immunogenicity from galcanezumab phase 3 trials in patients with episodic or chronic migraine. Cephalalgia.

[41] Bigal M.E., Walter S., Rapoport A.M. (2015). Therapeutic antibodies against CGRP or its receptor. Br. J. Clin. Pharmacol..

[42] Gklinos P., Papadopoulou M., Stanulovic V., Mitsikostas D.D., Papadopoulos D. (2021). Monoclonal antibodies as neurological therapeutics. Pharmaceuticals.

[43] Goadsby P.J., Dodick D.W., Leone M., Bardos J.N., Oakes T.M., Millen B.A., Zhou C., Dowsett S.A., Aurora S.K., Ahn A.H. (2019). Trial of galcanezumab in prevention of episodic cluster headache. N. Engl. J. Med..

[44] Förderreuther S., Zhang Q., Stauffer V.L., Aurora S.K., Láinez M.J.A. (2018). Preventive effects of galcanezumab in adult patients with episodic or chronic migraine are persistent: Data from the phase 3, randomized, double-blind, placebo-controlled EVOLVE-1, EVOLVE-2, and REGAIN studies. J. Headache Pain.

[45] Drellia. K., Kokoti L., Deligianni C.I., Papadopoulos D., Mitsikostas D.D. (2021). Anti-CGRP monoclonal antibodies for migraine prevention: A systematic review and likelihood to help or harm analysis. Cephalalgia.

[46] Pellesi L., Do T.P., Ashina H., Ashina M., Burstein R. (2020). Dual therapy with anti-CGRP monoclonal antibodies and botulinum toxin for migraine prevention: Is there a rationale?. Headache.

